# Potassium *N*,2-dichloro­benzene­sulfonamidate sesquihydrate

**DOI:** 10.1107/S1600536811021891

**Published:** 2011-06-11

**Authors:** B. Thimme Gowda, Sabine Foro, K. Shakuntala

**Affiliations:** a Department of Chemistry, Mangalore University, Mangalagangotri 574 199, Mangalore, India; b Institute of Materials Science, Darmstadt University of Technology, Petersenstrasse 23, D-64287 Darmstadt, Germany

## Abstract

In the title compound, K^+^·C_6_H_4_Cl_2_NO_2_S^−^·1.5H_2_O, one water mol­ecule has crystallographically imposed twofold symmetry. The K^+^ ion is heptacoordinated by three O atoms from water mol­ecules and by four sulfonyl O atoms of *N*-chloro-2-chloro-benzene­sulfonamide anions. The S—N distance of 1.582 (2) Å is consistent with an S—N double bond. In the structure, the sulfonyl-O and the water-O atoms bridge the K^+^ cations in a bidentate fashion. The crystal structure comprises sheets in the *ac* plane which are further stabilized by inter­molecular O—H⋯Cl and O—H⋯N hydrogen bonds.

## Related literature

For our studies of the effect of substituents on the structures of *N*-haloaryl­sulfonamides, see: Gowda *et al.* (2010 ▶, 2011**a* ▶,b* ▶); and on the oxidative strengths of *N*-haloaryl­sulfonamides, see: Gowda & Shetty (2004 ▶); Usha & Gowda (2006 ▶). For similar structures, see: George *et al.* (2000 ▶); Olmstead & Power (1986 ▶). For the preparation of the title compound, see: Jyothi & Gowda (2004 ▶).
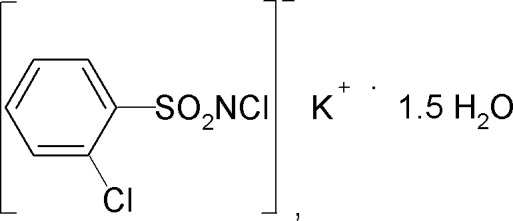


## Experimental

### 

#### Crystal data

K^+^·C_6_H_4_Cl_2_NO_2_S^−^·1.5H_2_O
*M* *_r_* = 291.19
Monoclinic, 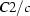

*a* = 12.301 (2) Å
*b* = 6.8277 (6) Å
*c* = 27.965 (3) Å
β = 106.28 (1)°
*V* = 2254.5 (5) Å^3^
*Z* = 8
Mo *K*α radiation
μ = 1.12 mm^−1^
*T* = 293 K
0.44 × 0.44 × 0.38 mm

#### Data collection

Oxford Diffraction Xcalibur diffractometer with Sapphire CCD detector
Absorption correction: multi-scan (*CrysAlis RED*; Oxford Diffraction, 2009 ▶) *T*_min_ = 0.640, *T*_max_ = 0.677
4174 measured reflections
2298 independent reflections
2181 reflections with *I* > 2σ(*I*)
*R*_int_ = 0.015

#### Refinement

*R*[*F* ^2^ > 2σ(*F* ^2^)] = 0.042
*wR*(*F* ^2^) = 0.105
*S* = 1.19
2298 reflections
142 parameters
3 restraints
H atoms treated by a mixture of independent and constrained refinement
Δρ_max_ = 0.47 e Å^−3^
Δρ_min_ = −0.46 e Å^−3^

### 

Data collection: *CrysAlis CCD* (Oxford Diffraction, 2009 ▶); cell refinement: *CrysAlis RED* (Oxford Diffraction, 2009 ▶); data reduction: *CrysAlis RED*; program(s) used to solve structure: *SHELXS97* (Sheldrick, 2008 ▶); program(s) used to refine structure: *SHELXL97* (Sheldrick, 2008 ▶); molecular graphics: *PLATON* (Spek, 2009 ▶); software used to prepare material for publication: *SHELXL97*.

## Supplementary Material

Crystal structure: contains datablock(s) I, global. DOI: 10.1107/S1600536811021891/sj5159sup1.cif

Structure factors: contains datablock(s) I. DOI: 10.1107/S1600536811021891/sj5159Isup2.hkl

Additional supplementary materials:  crystallographic information; 3D view; checkCIF report

## Figures and Tables

**Table 1:** Hydrogen-bond geometry (Å, °)

*D*—H⋯*A*	*D*—H	H⋯*A*	*D*⋯*A*	*D*—H⋯*A*
O3—H31⋯N1^i^	0.80 (2)	2.23 (2)	2.962 (3)	152 (4)
O3—H32⋯Cl1^ii^	0.81 (2)	2.73 (2)	3.517 (3)	165 (4)
O4—H41⋯N1^i^	0.81 (2)	2.19 (2)	2.978 (3)	165 (4)

Symmetry codes: (i) 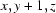; (ii) 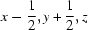.

**Fig. 1.**
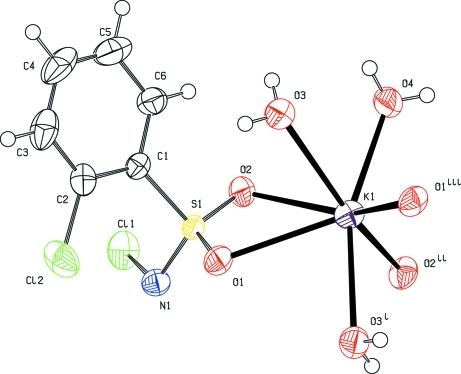
Molecular structure of the title compound, showing the atom labelling scheme for the asymmetric unit and extended to show the coordination geometry for the K+ cation. Displacement ellipsoids are drawn at the 50% probability level and H atoms are represented as small spheres of arbitrary radii (i) -x+1/2, y-1/2, -z+3/2; (ii) -x+1, y, -z+3/2; (iii) -x+1/2, y+1/2, -z+3/2.

**Fig. 2.**
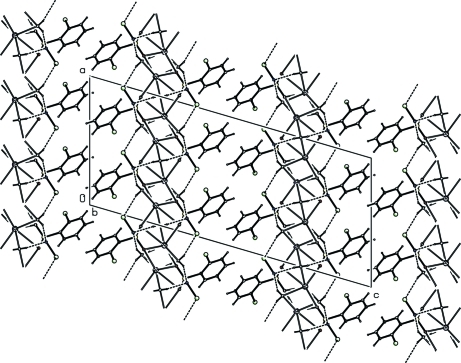
Molecular packing of the title compound with hydrogen bonds drawn as dashed lines.

## supplementary crystallographic information

### Comment

Arylsulfonamides and their N-halo compounds are of interest in synthetic, mechanistic and analytical chemistry (Gowda & Shetty, 2004; Usha & Gowda, 2006). To explore the substituent effects and the effect of replacing sodium ions by potassium ions on the solid state structures of *N*-halo- arylsulfonamides (Gowda *et al.*, 2010, 2011**a*,b*), in the present work, the structure of potassium *N*,2-dichloro-benzenesulfonamidate sesquihydrate (I) has been determined (Fig. 1). The structure of (I) is isostructural with potassium *N*-bromo-2-chloro-benzenesulfonamidate sesquihydrate (II) (Gowda *et al.*, 2011*b*), and resembles those of potassium *N*,4-dichloro-benzenesulfonamidate monohydrate (III) (Gowda *et al.*, 2011*a*), sodium *N*,2-dichloro-benzenesulfonamidate sesquihydrate (IV) (Gowda *et al.*, 2010) and other sodium *N*-chloro-arylsulfonamides (George *et al.*, 2000; Olmstead & Power, 1986).

In the title compound, K^+^ ion is hepta coordinated by three O atoms from water molecules and by four sulfonyl O atoms of *N*-chloro-2-chloro- benzenesulfonamidate anions. This is in contrast to hepta coordination of K^+^ by two O atoms from water molecules, four sulfonyl O atoms from the *N*-chloro-4-chlorobenzenesulfonamidate anions and one Cl in (III), and octahedral coordination of Na^+^ by three O atoms of water molecules and three sulfonyl O atoms of three different *N*-chloro-2-chloro- benzenesulfonamidate anions.

The S—N distance of 1.582 (4)Å is consistent with an S—N double bond and is in agreement with the observed values of 1.582 (4)Å in (II), 1.588 (2) Å in (III) and 1.588 (2) Å in (IV)

The crystal structure comprises sheets in the *ac* plane (Fig. 2). The molecular packing is stabilized by O3—H31···N1, O3—H32···Cl1 and O4—H41···N1 hydrogen bonds (Table 1).

### Experimental

The title compound was prepared by the method similar to that reported in literature (Jyothi & Gowda, 2004). The purity of the compound was checked by determining its melting point. Colourless prisms of (I) were obtained from its aqueous solution at room temperature.

### Refinement

The O bound H atoms were located in difference map and later restrained to O—H = 0.82 (2) Å The other H atoms were positioned with idealized geometry using a riding model with C—H = 0.93 Å. All H atoms were refined with isotropic displacement parameters (set to 1.2 times of the *U*_eq_ of the parent atom).

### Crystal data

**Table:**

K^+^·C_6_H_4_Cl_2_NO_2_S^−^·1.5H_2_O	*F*(000) = 1176
*M**_r_* = 291.19	*D*_x_ = 1.716 Mg m^−^^3^
Monoclinic, *C*2/*c*	Mo *K*α radiation, λ = 0.71073 Å
Hall symbol: -C 2yc	Cell parameters from 2350 reflections
*a* = 12.301 (2) Å	θ = 3.0–27.8°
*b* = 6.8277 (6) Å	µ = 1.12 mm^−^^1^
*c* = 27.965 (3) Å	*T* = 293 K
β = 106.28 (1)°	Prism, colourless
*V* = 2254.5 (5) Å^3^	0.44 × 0.44 × 0.38 mm
*Z* = 8	

### Data collection

**Table:**

Oxford Diffraction Xcalibur diffractometer with Sapphire CCD detector	2298 independent reflections
Radiation source: fine-focus sealed tube	2181 reflections with *I* > 2σ(*I*)
graphite	*R*_int_ = 0.015
ω scans.	θ_max_ = 26.4°, θ_min_ = 3.0°
Absorption correction: multi-scan (*CrysAlis RED*; Oxford Diffraction, 2009)	*h* = −15→10
*T*_min_ = 0.640, *T*_max_ = 0.677	*k* = −6→8
4174 measured reflections	*l* = −34→34

### Refinement

**Table:**

Refinement on *F*^2^	Secondary atom site location: difference Fourier map
Least-squares matrix: full	Hydrogen site location: inferred from neighbouring sites
*R*[*F*^2^ > 2σ(*F*^2^)] = 0.042	H atoms treated by a mixture of independent and constrained refinement
*wR*(*F*^2^) = 0.105	*w* = 1/[σ^2^(*F*_o_^2^) + (0.0457*P*)^2^ + 5.0762*P*] where *P* = (*F*_o_^2^ + 2*F*_c_^2^)/3
*S* = 1.19	(Δ/σ)_max_ = 0.002
2298 reflections	Δρ_max_ = 0.47 e Å^−^^3^
142 parameters	Δρ_min_ = −0.46 e Å^−^^3^
3 restraints	Extinction correction: *SHELXL*, Fc^*^=kFc[1+0.001xFc^2^λ^3^/sin(2θ)]^-1/4^
Primary atom site location: structure-invariant direct methods	Extinction coefficient: 0.0234 (10)

### Special details

**Table:**

Geometry. All e.s.d.'s (except the e.s.d. in the dihedral angle between two l.s. planes) are estimated using the full covariance matrix. The cell e.s.d.'s are taken into account individually in the estimation of e.s.d.'s in distances, angles and torsion angles; correlations between e.s.d.'s in cell parameters are only used when they are defined by crystal symmetry. An approximate (isotropic) treatment of cell e.s.d.'s is used for estimating e.s.d.'s involving l.s. planes.
Refinement. Refinement of *F*^2^ against ALL reflections. The weighted *R*-factor *wR* and goodness of fit *S* are based on *F*^2^, conventional *R*-factors *R* are based on *F*, with *F* set to zero for negative *F*^2^. The threshold expression of *F*^2^ > σ(*F*^2^) is used only for calculating *R*-factors(gt) *etc*. and is not relevant to the choice of reflections for refinement. *R*-factors based on *F*^2^ are statistically about twice as large as those based on *F*, and *R*- factors based on ALL data will be even larger.

### Fractional atomic coordinates and isotropic or equivalent isotropic displacement parameters (Å^2^)

**Table:**

	*x*	*y*	*z*	*U*_iso_*/*U*_eq_	
C1	0.2790 (2)	0.2499 (4)	0.60429 (9)	0.0268 (5)	
C2	0.1789 (2)	0.1729 (4)	0.57347 (10)	0.0346 (6)	
C3	0.1218 (3)	0.2695 (6)	0.53041 (12)	0.0510 (8)	
H3	0.0551	0.2173	0.5100	0.061*	
C4	0.1633 (4)	0.4426 (6)	0.51767 (13)	0.0599 (10)	
H4	0.1241	0.5075	0.4887	0.072*	
C5	0.2620 (3)	0.5203 (5)	0.54735 (13)	0.0545 (9)	
H5	0.2899	0.6373	0.5384	0.065*	
C6	0.3204 (3)	0.4242 (4)	0.59073 (11)	0.0383 (6)	
H6	0.3874	0.4769	0.6108	0.046*	
N1	0.38459 (19)	−0.0793 (3)	0.65478 (9)	0.0328 (5)	
O1	0.27693 (18)	0.1323 (3)	0.69244 (7)	0.0380 (5)	
O2	0.45006 (17)	0.2695 (3)	0.68135 (7)	0.0402 (5)	
O3	0.2321 (2)	0.6414 (3)	0.68516 (8)	0.0431 (5)	
H31	0.260 (3)	0.704 (5)	0.6675 (12)	0.052*	
H32	0.181 (3)	0.584 (5)	0.6658 (12)	0.052*	
O4	0.5000	0.7199 (5)	0.7500	0.0478 (8)	
H41	0.477 (3)	0.791 (5)	0.7260 (10)	0.057*	
K1	0.35556 (5)	0.42956 (9)	0.76528 (2)	0.0342 (2)	
Cl1	0.48019 (7)	−0.09326 (13)	0.61910 (3)	0.0478 (2)	
Cl2	0.12087 (7)	−0.04411 (13)	0.58723 (4)	0.0561 (3)	
S1	0.35458 (5)	0.14187 (9)	0.66219 (2)	0.0262 (2)	

### Atomic displacement parameters (Å^2^)

**Table:**

	*U*^11^	*U*^22^	*U*^33^	*U*^12^	*U*^13^	*U*^23^
C1	0.0265 (11)	0.0307 (13)	0.0240 (11)	0.0051 (10)	0.0085 (9)	0.0003 (10)
C2	0.0302 (13)	0.0381 (14)	0.0329 (13)	0.0059 (11)	0.0047 (11)	−0.0060 (11)
C3	0.0414 (17)	0.067 (2)	0.0359 (16)	0.0174 (16)	−0.0028 (13)	−0.0059 (15)
C4	0.072 (2)	0.069 (2)	0.0342 (17)	0.032 (2)	0.0082 (16)	0.0163 (16)
C5	0.071 (2)	0.0499 (19)	0.0461 (18)	0.0114 (17)	0.0224 (17)	0.0192 (15)
C6	0.0433 (16)	0.0370 (15)	0.0360 (14)	0.0002 (12)	0.0133 (12)	0.0035 (12)
N1	0.0325 (12)	0.0327 (12)	0.0339 (12)	0.0018 (9)	0.0104 (9)	0.0021 (9)
O1	0.0449 (11)	0.0444 (11)	0.0309 (10)	−0.0048 (9)	0.0208 (9)	−0.0007 (8)
O2	0.0373 (11)	0.0430 (11)	0.0341 (10)	−0.0127 (9)	−0.0002 (8)	−0.0018 (9)
O3	0.0455 (12)	0.0427 (12)	0.0387 (12)	−0.0017 (10)	0.0079 (9)	0.0016 (9)
O4	0.0565 (19)	0.0343 (16)	0.0420 (17)	0.000	−0.0038 (15)	0.000
K1	0.0325 (3)	0.0354 (3)	0.0366 (3)	0.0065 (2)	0.0129 (2)	0.0028 (2)
Cl1	0.0399 (4)	0.0590 (5)	0.0472 (4)	0.0057 (3)	0.0164 (3)	−0.0102 (4)
Cl2	0.0364 (4)	0.0480 (5)	0.0718 (6)	−0.0112 (3)	−0.0049 (4)	−0.0054 (4)
S1	0.0271 (3)	0.0299 (3)	0.0206 (3)	−0.0043 (2)	0.0053 (2)	0.0000 (2)

### Geometric parameters (Å, °)

**Table:**

C1—C6	1.388 (4)	O1—K1	2.846 (2)
C1—C2	1.393 (4)	O2—S1	1.440 (2)
C1—S1	1.785 (2)	O2—K1^ii^	2.672 (2)
C2—C3	1.380 (4)	O2—K1	3.096 (2)
C2—Cl2	1.734 (3)	O3—K1	2.741 (2)
C3—C4	1.373 (6)	O3—K1^iii^	2.791 (2)
C3—H3	0.9300	O3—H31	0.797 (19)
C4—C5	1.372 (6)	O3—H32	0.806 (19)
C4—H4	0.9300	O4—K1	2.774 (2)
C5—C6	1.389 (4)	O4—K1^ii^	2.774 (2)
C5—H5	0.9300	O4—H41	0.811 (18)
C6—H6	0.9300	K1—O1^iii^	2.655 (2)
N1—S1	1.582 (2)	K1—O2^ii^	2.672 (2)
N1—Cl1	1.745 (2)	K1—O3^i^	2.791 (2)
O1—S1	1.4442 (19)	K1—S1	3.4859 (9)
O1—K1^i^	2.655 (2)		
			
C6—C1—C2	118.9 (2)	O2^ii^—K1—O3	151.25 (7)
C6—C1—S1	117.7 (2)	O1^iii^—K1—O4	101.28 (7)
C2—C1—S1	123.4 (2)	O2^ii^—K1—O4	82.34 (6)
C3—C2—C1	120.3 (3)	O3—K1—O4	74.28 (6)
C3—C2—Cl2	117.5 (2)	O1^iii^—K1—O3^i^	77.05 (7)
C1—C2—Cl2	122.2 (2)	O2^ii^—K1—O3^i^	81.03 (7)
C4—C3—C2	120.2 (3)	O3—K1—O3^i^	125.11 (4)
C4—C3—H3	119.9	O4—K1—O3^i^	158.97 (5)
C2—C3—H3	119.9	O1^iii^—K1—O1	124.24 (4)
C5—C4—C3	120.4 (3)	O2^ii^—K1—O1	100.34 (7)
C5—C4—H4	119.8	O3—K1—O1	77.99 (7)
C3—C4—H4	119.8	O4—K1—O1	120.21 (5)
C4—C5—C6	119.9 (3)	O3^i^—K1—O1	75.62 (6)
C4—C5—H5	120.0	O1^iii^—K1—O2	158.33 (6)
C6—C5—H5	120.0	O2^ii^—K1—O2	79.15 (7)
C1—C6—C5	120.3 (3)	O3—K1—O2	78.84 (6)
C1—C6—H6	119.9	O4—K1—O2	75.08 (6)
C5—C6—H6	119.9	O3^i^—K1—O2	114.01 (7)
S1—N1—Cl1	110.28 (13)	O1—K1—O2	47.94 (5)
S1—O1—K1^i^	150.89 (13)	O1^iii^—K1—S1	143.30 (5)
S1—O1—K1	103.85 (10)	O2^ii^—K1—S1	91.52 (5)
K1^i^—O1—K1	100.43 (6)	O3—K1—S1	75.44 (5)
S1—O2—K1^ii^	164.66 (13)	O4—K1—S1	97.42 (4)
S1—O2—K1	93.17 (10)	O3^i^—K1—S1	95.81 (5)
K1^ii^—O2—K1	84.23 (6)	O1—K1—S1	23.72 (4)
K1—O3—K1^iii^	99.72 (7)	O2—K1—S1	24.36 (4)
K1—O3—H31	124 (3)	O2—S1—O1	114.44 (12)
K1^iii^—O3—H31	103 (3)	O2—S1—N1	115.50 (13)
K1—O3—H32	116 (3)	O1—S1—N1	104.51 (12)
K1^iii^—O3—H32	110 (3)	O2—S1—C1	104.51 (12)
H31—O3—H32	103 (4)	O1—S1—C1	106.75 (12)
K1—O4—K1^ii^	88.78 (10)	N1—S1—C1	110.93 (12)
K1—O4—H41	118 (3)	O2—S1—K1	62.47 (9)
K1^ii^—O4—H41	113 (3)	O1—S1—K1	52.43 (9)
O1^iii^—K1—O2^ii^	121.97 (7)	N1—S1—K1	134.37 (9)
O1^iii^—K1—O3	79.63 (7)	C1—S1—K1	113.42 (8)
			
C6—C1—C2—C3	−0.4 (4)	K1—O2—S1—O1	−7.24 (13)
S1—C1—C2—C3	176.7 (2)	K1^ii^—O2—S1—N1	−49.0 (5)
C6—C1—C2—Cl2	179.9 (2)	K1—O2—S1—N1	−128.69 (10)
S1—C1—C2—Cl2	−3.0 (3)	K1^ii^—O2—S1—C1	−171.1 (5)
C1—C2—C3—C4	−0.1 (5)	K1—O2—S1—C1	109.15 (9)
Cl2—C2—C3—C4	179.6 (3)	K1^ii^—O2—S1—K1	79.7 (5)
C2—C3—C4—C5	0.5 (5)	K1^i^—O1—S1—O2	−137.6 (2)
C3—C4—C5—C6	−0.4 (6)	K1—O1—S1—O2	8.11 (15)
C2—C1—C6—C5	0.6 (4)	K1^i^—O1—S1—N1	−10.2 (3)
S1—C1—C6—C5	−176.7 (2)	K1—O1—S1—N1	135.42 (10)
C4—C5—C6—C1	−0.2 (5)	K1^i^—O1—S1—C1	107.3 (3)
K1^iii^—O3—K1—O1^iii^	16.67 (7)	K1—O1—S1—C1	−106.99 (11)
K1^iii^—O3—K1—O2^ii^	−125.10 (13)	K1^i^—O1—S1—K1	−145.7 (3)
K1^iii^—O3—K1—O4	−88.29 (7)	Cl1—N1—S1—O2	−52.68 (17)
K1^iii^—O3—K1—O3^i^	82.64 (12)	Cl1—N1—S1—O1	−179.33 (13)
K1^iii^—O3—K1—O1	145.25 (8)	Cl1—N1—S1—C1	65.98 (16)
K1^iii^—O3—K1—O2	−165.75 (8)	Cl1—N1—S1—K1	−128.22 (10)
K1^iii^—O3—K1—S1	169.49 (7)	C6—C1—S1—O2	−2.1 (2)
K1^ii^—O4—K1—O1^iii^	164.53 (5)	C2—C1—S1—O2	−179.2 (2)
K1^ii^—O4—K1—O2^ii^	43.34 (5)	C6—C1—S1—O1	119.5 (2)
K1^ii^—O4—K1—O3	−119.75 (6)	C2—C1—S1—O1	−57.6 (2)
K1^ii^—O4—K1—O3^i^	81.31 (19)	C6—C1—S1—N1	−127.2 (2)
K1^ii^—O4—K1—O1	−54.21 (6)	C2—C1—S1—N1	55.7 (2)
K1^ii^—O4—K1—O2	−37.41 (4)	C6—C1—S1—K1	63.8 (2)
K1^ii^—O4—K1—S1	−47.208 (16)	C2—C1—S1—K1	−113.3 (2)
S1—O1—K1—O1^iii^	149.55 (9)	O1^iii^—K1—S1—O2	143.82 (13)
K1^i^—O1—K1—O1^iii^	−46.65 (10)	O2^ii^—K1—S1—O2	−58.97 (8)
S1—O1—K1—O2^ii^	−69.62 (12)	O3—K1—S1—O2	95.08 (11)
K1^i^—O1—K1—O2^ii^	94.17 (7)	O4—K1—S1—O2	23.50 (11)
S1—O1—K1—O3	81.10 (11)	O3^i^—K1—S1—O2	−140.10 (11)
K1^i^—O1—K1—O3	−115.10 (8)	O1—K1—S1—O2	−171.67 (15)
S1—O1—K1—O4	17.48 (14)	O1^iii^—K1—S1—O1	−44.51 (11)
K1^i^—O1—K1—O4	−178.72 (6)	O2^ii^—K1—S1—O1	112.70 (12)
S1—O1—K1—O3^i^	−147.47 (12)	O3—K1—S1—O1	−93.24 (12)
K1^i^—O1—K1—O3^i^	16.33 (7)	O4—K1—S1—O1	−164.82 (12)
S1—O1—K1—O2	−4.61 (8)	O3^i^—K1—S1—O1	31.57 (12)
K1^i^—O1—K1—O2	159.19 (11)	O2—K1—S1—O1	171.67 (15)
K1^i^—O1—K1—S1	163.80 (15)	O1^iii^—K1—S1—N1	−116.44 (14)
S1—O2—K1—O1^iii^	−72.9 (2)	O2^ii^—K1—S1—N1	40.78 (13)
K1^ii^—O2—K1—O1^iii^	122.30 (16)	O3—K1—S1—N1	−165.17 (13)
S1—O2—K1—O2^ii^	119.28 (6)	O4—K1—S1—N1	123.25 (13)
K1^ii^—O2—K1—O2^ii^	−45.55 (9)	O3^i^—K1—S1—N1	−40.36 (13)
S1—O2—K1—O3	−79.32 (10)	O1—K1—S1—N1	−71.93 (16)
K1^ii^—O2—K1—O3	115.85 (7)	O2—K1—S1—N1	99.75 (16)
S1—O2—K1—O4	−155.84 (10)	O1^iii^—K1—S1—C1	49.10 (13)
K1^ii^—O2—K1—O4	39.33 (5)	O2^ii^—K1—S1—C1	−153.68 (10)
S1—O2—K1—O3^i^	44.31 (12)	O3—K1—S1—C1	0.37 (11)
K1^ii^—O2—K1—O3^i^	−120.52 (7)	O4—K1—S1—C1	−71.21 (10)
S1—O2—K1—O1	4.50 (8)	O3^i^—K1—S1—C1	125.18 (10)
K1^ii^—O2—K1—O1	−160.33 (10)	O1—K1—S1—C1	93.61 (15)
K1^ii^—O2—K1—S1	−164.83 (13)	O2—K1—S1—C1	−94.71 (13)
K1^ii^—O2—S1—O1	72.5 (5)		

Symmetry codes: (i) −*x*+1/2, *y*−1/2, −*z*+3/2; (ii) −*x*+1, *y*, −*z*+3/2; (iii) −*x*+1/2, *y*+1/2, −*z*+3/2.

### Hydrogen-bond geometry (Å, °)

**Table:**

*D*—H···*A*	*D*—H	H···*A*	*D*···*A*	*D*—H···*A*
O3—H31···N1^iv^	0.80 (2)	2.23 (2)	2.962 (3)	152 (4)
O3—H32···Cl1^v^	0.81 (2)	2.73 (2)	3.517 (3)	165 (4)
O4—H41···N1^iv^	0.81 (2)	2.19 (2)	2.978 (3)	165 (4)

Symmetry codes: (iv) *x*, *y*+1, *z*; (v) *x*−1/2, *y*+1/2, *z*.
